# Supplementary material for: The Construction and Exploration of a Comprehensive MicroRNA Centered Regulatory Network in Foxtail Millet (Setaria italica L.)
Source: Front Plant Sci. 2022 May 6;13:848474. doi: 10.3389/fpls.2022.848474 (PMC9121102; doi:10.3389/fpls.2022.848474)
Supplement: Supplementary file 6 [file Data_Sheet_6.zip › Sit-miRNovel4_Seita.5G154800.1_1213_TPlot.pdf]

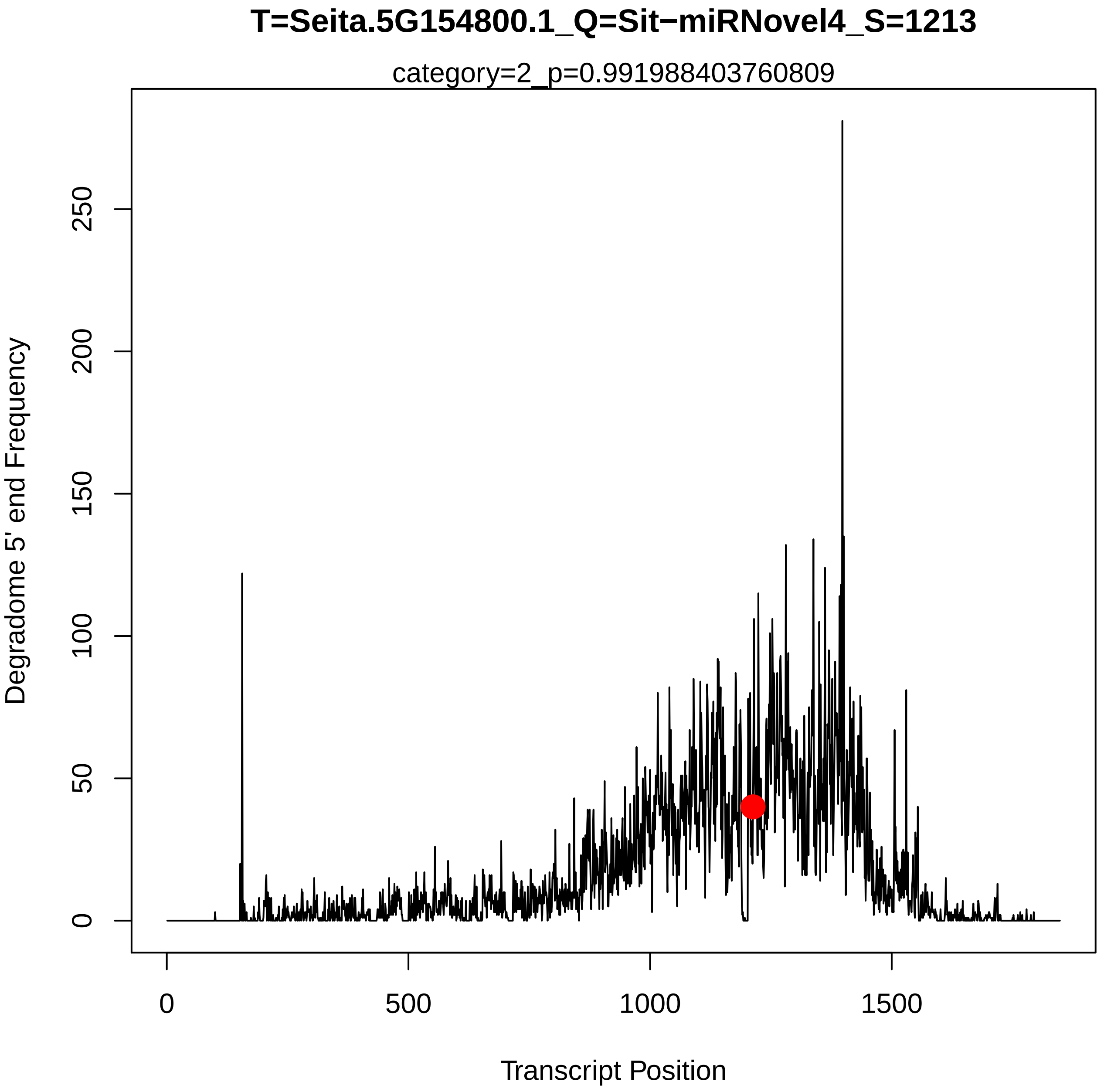
T=Seita.5G154800.1_Q=Sit−miRNovel4_S=1213
category=2_p=0.991988403760809
Degradome 5' end Frequency
0
50
100
150
200
250
Transcript Position
0
500
1000
1500
